# Protein disorder - function paradigm: Putative role in inflammation

**DOI:** 10.6026/973206300210169

**Published:** 2025-02-28

**Authors:** Francesco Chiappelli

**Affiliations:** 1 Dental Group of Sherman Oaks, Sherman Oaks, California-91403; 2 UCLA Center for the Health Sciences, Los Angeles, California-90095

**Keywords:** Disorder-function paradigm, intrinsically disordered proteins (IDPs), dark proteome, immunome, inflammasome, meta-inflammation, inflammAging, cGAS-STING, AI

## Abstract

Traditional protein biochemistry defends the intimate interdependence between protein function and structure, the latter being consisting of four distinct levels: premary sstructure: *viz,* the sequence of its constituent amino acids linked by peptides bonds - the polypeptide chain; ssecondary structure: *viz,* localized folding patterns (*e.g.*, a α-helix, β-sheets) of the polypeptide chain held by hydrogen bonds between amino acid backbones; tertiary structure: *viz,* three-dimensional folding of the protein held by interactions between amino acid side chains mediated by disulfide bridges, hydrogen bonds, ionic bonds and hydrophobic interactions; and quaternary structure: *viz,* attachment of subunits, when appropriate, by means of similar chemical interactions to form a functional protein complex. Research evidence in the last decade has described intrinsically disordered proteins (IDPs) as polypeptides that lack a well-defined three/four-dimensional structure under physiological conditions, appear structurally unstable and manifest in a dynamic set of possible conformations. IDPs are a major component of the "dark" proteome, genome protein products not yet characterized through experimental structure determination and existing homology modeling. Dark proteome in general and IDPs specifically define and characterize the novel disorder - function paradigm, which critically mediate and modulate key cellular organelles and pathways and influence physiopathological processes from aging to chronic diseases and pathogen infection. The role of the disorder-function paradigm in the immunome and the inflammasome in general and specifically in the process of chronic metabolic inflammation observed in aging - *i.e.*, inflammAging - could be elucidated through diverse AI platforms.

## Background:

## The disorder-function paradigm:

The "disorder-function paradigm" of proteins defines and characterizes the spectrum of the various facets of intrinsically disordered proteins (IDPs) and IDP regions. To be clear, several proteins and protein segments, products of the dark proteome, fail to attain a single stable three/four-dimensional structure under physiological conditions, but rather manifest as multiple interconverting conformational states [1]. These fluid structure-function conditions can affect a variety of polypeptides and polypeptide regions with critical cellular roles and are therefore increasingly viewed as critical to metabolism and physiological regulation, particularly, but not limited to the domain of transmembrane proteins [2, 3].

Case in point, the four members of the epidermal growth factor receptor family designated after the erythroblast leukemia viral oncogene (ErbB) - *viz.*, ErbB1-4. These specialized proteinic products are receptor tyrosine kinases that modulate critical steps in development, normal physiology and across a wide variety of pathological states, from autoimmune diseases (*e.g.*, multiple sclerosis to malignancies. When tyrosine-phosphorylated, activated dimers of ErbBs are formed and their C-terminals behave characteristically as IDP regions to regulate kinase function [4, 5]. Beyond coordinating certain signaling events, modulating gene expression and regulating ion channels, structural/functional IDPs and IDP regions impact fundamental domains of structural biology [6], which can be elucidated or predicted in part by AI [7, 8]. Therefore, the "disorder-function paradigm" appears crucial to characterizing the flexible structure and ability of IDPs and IDP regions to bind to a multitude of surfaces, including minerals and crystals - *viz,* the process of bio mineralization [9] for the formation of teeth, bones and other mineralized tissues in health (*e.g.*, anchoring muscles and ligaments to bones) and disease (*e.g.*, calcification of cardiovascular tissues).

## IDPs modulation of acute inflammation and infection:

The "disorder-function paradigm" that involves IDPs and IDP regions, also plays a critical role in pathogen binding and infection. For example, a specific IDP region from glutaredoxin residue modulates the binding of the pathogenic *Trypanosoma brucei* parasite, causative factor for the sleeping sickness [10]. Similarly, the translocate actin recruiting phosphoprotein expressed by chlamydial species is an IDP effector protein that remodels the host-actin cytoskeleton during the initial stage of that bacterial infection [11]. Moreover, several, if not most DNA and RNA viruses initiate host cell binding and infection through interactions with host membrane proteins by binding viral IDP regions to host receptor globular domains [12, 13]. To be clear, pathogens often employ IDPs or IPD regions that mimic eukaryotic linear motif to perturb and hijack host cell networks to ensure productive infection [14] and impair, if not blunt innate and adaptive immune surveillance at the cellular and the humoral levels [15].

The initial immune response to parasitic, bacterial or viral pathogens is an acute inflammatory response [16, 17]. That is to say, certain cytosolic pattern-recognition receptors [15] form multi-protein complexes, referred to as canonical inflammasome in response to pathogens. Canonical inflammasome then recruit and activate caspase-1, formerly known as the interleukin-1 converting enzyme, which cleaves and activates the inflammatory cytokines interleukin 1β and interleukin 18 into their active forms [18]. Indeed, viral DNA or RNA and viral proteins and proteases often carry IDP regions that interfere with, or modulate the inflammasome [19] and the same can possibly are argued for bacterial nucleic acid proteinic products. Moreover, the acute inflammatory response can become persistent, with consequential onset of the catabolism syndrome, if the presence of the noxious agent persists [20].

## Chronic metabolic inflammation in health and disease:

By contrast, chronic metabolic inflammation is characterized by a systemic state of persistent inflammation in the absence of a triggering pathogen. It is a sustained long-term physiological state of altered homeostasis, of sterile long-term metabolic inflammation that reflects directly and unambiguously the bilateral crosstalk between immune regulation and aberrant metabolism, the interface of which is a persistent inflammatory state in the absence of infectious pathogen or other physical triggers [21, 22]. Metabolic inflammation brings about significant pathological *Sequelae* that impair immunity and that represents the leading causes of disability and mortality from cardiovascular disease, cancer, diabetes mellitus, metabolic syndrome, chronic kidney disease, non-alcoholic fatty liver disease and autoimmune and neurodegenerative disorders globally. Advancing age is also associated with a state of chronic metabolic inflammation, termed inflammAging [22, 23].

Evidence suggests that inflammAging results in large part from deregulated metabolic interference brought about by a chronic state of low-level inflammation. It is believed to involve the cGAS-STING signaling pathway, which mediates immune sensing of cytosolic DNA, such as that released by lyses bacteria of replicating viruses [24]. In brief, foreign or intrusive cytosolic DNA and related nucleic acids are detected and recognized by the cell as potentially pathogenic, engendering an interferon-related response that induces the stimulator of interferon genes (STING) via the activation of the cyclic GMP-AMP (cGMP-AMP) synthase enzyme (cGAS). CGAS-mediated activation drives the translocation of STING to the endoplasmic reticulum/Golgi apparatus interface, where it recruits the serine/threonine kinase tumor necrosis factor receptor-associated factor (TRAF)-associated NFκB activator (TANK) binding kinase 1 (TBK1). The STING-TBK1 complex is then trafficked into the Golgi apparatus by means of the Coat Protein Complex II (COPII) to facilitate the formation of vesicles that transport foreign materials, such as trunked pieces of foreign nucleic acids and proteins, from the endoplasmic reticulum and the translocon-associated protein beta (TRAPβ), a proteinic complex required for protein translocation across the endoplasmic reticulum membrane following translation [25]. In brief, the cGAS-STING axis, in and of itself rich in IPD regions, is a physiologic regulatory process specialized to link the sensing the abnormal presence of DNA and other nucleic acids in cellular compartments to the immune inflammatory response. It is generally beneficial, but excessive engagement of cGAS-STING, such as is the case in inflammAging and impairs homeostasis and immunity [26].

## The disorder-function paradigm in inflammaging:

In conclusion, the immunome [15], the cellular and molecular immune system parameters that define and characterize the individual response of the immune system to pathogens as determined by a spectrum of individualized parameters that range from the microenvironment [27], the fractalomic idio type/anti-idiotypic profile [28], to the allostasiome [29] and the inflammasome [15], the spectrum of key inflammatory and anti-inflammatory regulators of the host in response to threat, injury or microbial pathogen infection, during the aging process can best be defined and characterized by Machine Learning [28, 30] and other AI platforms [31]. As also noted above, foreign nucleic acid and proteinic products released by pathogenic viruses, bacteria and parasites can trigger cGAS [19] via their IDP regions to modulate cGAS-STING and playing a pivotal role in the innate immune response and pathogenesis of various diseases [32]. It follows that cGAS-STING and the cGAS-STING-dependent aging-related endothelial dysfunction [33] and accelerated senescence of pancreatic β-cells [34], may be among the pathways, if not one of the principal pathways through which the "disorder-function paradigm" - *viz,* the universe of IDPs and IDP regions - contributes to the broad immunopathology of inflammAging across its multiple immune and metabolic diseases [35].

## Declaration on publication ethics:

The author's state that they adhere with COPE guidelines on publishing ethics as described elsewhere at https://publicationethics.org/. The authors also undertake that they are not associated with any other third party (governmental or non-governmental agencies) linking with any form of unethical issues connecting to this publication. The authors also declare that they are not withholding any information that is misleading to the publisher in regard to this article.

## Declaration on official E-mail:

The corresponding author declares that lifetime official e-mail from their institution is not available for all authors

## License statement:

This is an Open Access article which permits unrestricted use, distribution and reproduction in any medium, provided the original work is properly credited. This is distributed under the terms of the Creative Commons Attribution License.

## Comments from readers:

Articles published in BIOINFORMATION are open for relevant post publication comments and criticisms, which will be published immediately linking to the original article without open access charges. Comments should be concise, coherent and critical in less than 1000 words.
